# A Case in Point

**Published:** 1888-01

**Authors:** 


### A CASE IN POINT.

We have received the following postal from one of our old-time patrons, which encourages us to believe we are doing some good in our limited sphere of action.

Y. M. C. A. BUTLER, PA. Dec. 13, 1887.

"When our subscription to the Hall's Journal of Health expires, please discontinue it. Very Truly, J. B. QUINN, *General Secretary.*

We find no fault with the above request. It is brief, courteous and to the point. The fault if any there be is doubtless ours. For a period of two years it has been our constant endeavor to bring to the knowledge of our readers as a substitute for some crude and anti-christian doctrines which so called Christian Associations have been *forced* to relinquish, more humane and reasonable views of the author of Being, His institutions and laws as related to man's responsibility, accountability and ultimate destiny. With all enlightened individuals and communities, the story of the creation and fall of man, of original sin, the atonement and possibility of redemption through the shedding of innocent blood, have been relegated to the region of myths and bugbears, from which they were incorporated into senseless creeds and held by a cunning priesthood *in terrorem* over ignorant and superstitious flocks, whose lives were tortured, hearts sickened and death-beds harrassed by false conceptions of a beneficent Creator, to whom were attributed qualities as cruel, revengful and merciless as the worst elements of an imaginary satan. We say to the suffering look upward, to the afflicted take heart and to the dying be of good cheer, for the God of the Universe is a God of love, of justice and unfaltering mercy. Those persons and associations who deem such words an attack upon their religion, ought to refuse Hall's Journal of Health, which out of a solemn duty to the afflicted is bound to expose the borrowed myths, dogmas and superstitions which have become incorporated into popular beliefs, wherein the Divine Ruler of all things, is presented in the likeness of man, with all human passions and imperfections magnified, besides having set him up a rival with too small a difference to make a vivid contrast.

"All are but parts of one stupendous whole
Whose body Nature is, and God the Soul."

